# Comparison of Extracorporeal Shock Wave Lithotripsy for Urolithiasis Between Children and Adults: A Single Centre Study

**DOI:** 10.7759/cureus.810

**Published:** 2016-09-29

**Authors:** Nadeem Iqbal, Salman Assad, Joshua Rahat Aleman Bhatti, Aisha Hasan, Muhammad Usman Shabbir, Saeed Akhter

**Affiliations:** 1 `Department of Urology, Shifa International Hospital, Islamabad, Pakistan; 2 Department of Neurology & Neurosurgery, Shifa Tameer-e-Millat University, Islamabad, Pakistan; 3 Department of Urology, Shifa International Hospital, Islamabad, Pakistan; 4 Department of Cardiology, Shifa International Hospital, Islamabad, Pakistan

**Keywords:** eswl, adults, pediatric, complication, stone free rate

## Abstract

**Objective:**

To retrospectively evaluate the effectiveness of extracorporeal shock wave lithotripsy (ESWL) for urolithiasis and compare the results between children and adults.

**Materials and methods:**

From January 2011 to January 2015 (four years), ESWL was performed in 104 children and 300 adults for urolithiasis. MODULITH^® ^SLX-F2 lithotripter (Storz Medical AG, Tägerwilen, Switzerland) equipment was used for ESWL. The stone-free rates, the number of ESWL sessions required, complication rates and ancillary procedures used were evaluated in a comparative manner.

**Results:**

The mean age ± standard deviation (SD) of children was 7.84±4.22 years and of adults was a 40.22±1.57 years. Mean ± SD of the stone size was 1.28±61 cm in the adults while 1.08 ± 0.59 cm in the children. In adults, the complications included steinstrasse in six (1.98%) patients, fever in 15 (4.95%), hematuria in 19 (6.28%) and sepsis in six (1.98%) patients. In children, steinstrasse was observed in two (1.9%), mild fever in two (1.9%), hematuria in six (5.7%) and sepsis was seen in four (3.8%) patients. The overall complication rate in the adults and in the children, it was found to be 46/300 (15%) and in the children, it was seen to be 14/104 (13%). No statistical difference was found in post-ESWL complications between children and adults (P>0.05). Ancillary procedures including double J (DJ) stent were used in 13 (12.5%) children and 87 (29%) adults. There was a better stone clearance rate in children i.e. 79% as compared to 68% in adults (*X^2^: *P=0.036).

**Conclusion:**

Children can achieve high stone-free rates after ESWL with a lower need for repeat ancillary procedures as compared to adults. However, there is a difference in the post-ESWL complications between these groups.

## Introduction

Extracorporeal shock wave lithotripsy (ESWL) has altered the treatment of upper tract stones in children [1]. Extracorporeal shock wave lithotripsy is harmless, safe and effective with excellent stone clearance rates in children [2-4]. The success rates and complications after ESWL are determined by size, location, composition stones, anatomical features of the urinary tract and the type of lithotripter used [5]. The complications that occur after extracorporeal shock wave lithotripsy include steinstrasse that may result due to impacted fragments in the ureter, but no concordance has been achieved such that ureteric stenting could be used to avoid steinstrasse and other post-ESWL complications [6]. While choosing an appropriate treatment approach to urolithiasis, certain parameters in consideration are the number of stones and their size, composition, location and presence of hydronephrosis, and other anatomical factors such as ureteric anomalies, the presence of a solitary kidney, strictures and morbid obesity [7-8]. Regarding the age factor, it has been noted that stone-free rate in children who underwent ESWL was greater as compared to that in adults [9]. This higher stone-free rate in children has been attributed to the small body volume allows shockwave transmission with minimal loss of energy [10]. The minimally invasive approach of ESWL is favored in children owing to the smaller size of the ureter and urethra [11]. But there is also a problem of the much extra burden of the probable use of general anesthesia in children especially when multiple sessions of ESWL are required. ESWL is the effective treatment for urolithiasis in both adults and children [12]. However, our study focuses on whether the efficacy of extracorporeal shock wave lithotripsy is more in children as compared to adults or not. 

## Materials and methods

**Figure 1:**
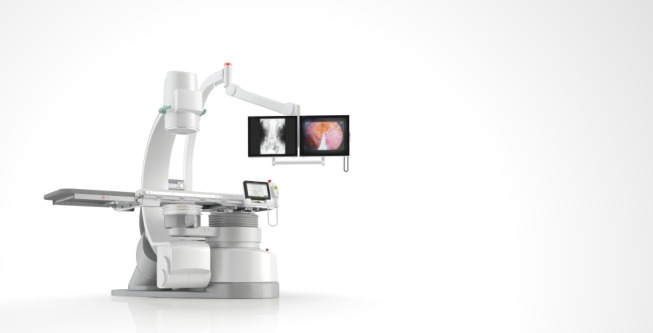
MODULITH® SLX-F2 lithotripter STORZ medical equipment

From January 2011 to January 2015, ESWL was performed on 104 children aged ≤ 17 years and 300 adults aged > 17 years for urolithiasis. MODULITH^® ^SLX-F2 lithotripter STORZ medical equipment was used for ESWL (Figure 1). The study was conducted at a tertiary care Shifa International Hospital, Islamabad, Pakistan. The stone-free rates, the number of ESWL sessions required, complication rates and ancillary procedures used were evaluated in a comparative manner. The exclusion criteria consisted of stones of ≥ 2 cm with the longest diameter, pregnant women, urinary tract infection with fever, bleeding diathesis, and malfunctioned kidneys. We did a complete blood count, urine culture, coagulation profile, ultrasonography and KUB (kidney ureter and bladder) CT scan for all patients.

During ESWL, the energy level used for adult kidney stones varied between 5-7 kV, and for ureteric stones, it was between 5-9 kV. Some shocks per session were 3500 for kidney and 4000 for ureter in the adult group. The energy level was increased gradually. In the pediatric group the energy level for kidney stones was kept at 2 to 4 kV, while for ureteric stones it was kept between 3-5 kV kilovolts. Some shocks per session were 2500 for kidney and 3000 for ureter in the pediatric group. The time of the procedure was approximately 45 minutes to 55 minutes in adults, while up to 45 minutes in children. In adults, the procedure was performed after obtaining consent from the patient. They were fully informed about the possible success rates and the complications during or after the procedure. In the pediatric group, consent was taken from the parents after thorough counseling. In the children below ten years of general age, anesthesia was used. In the children aged above ten years of age dissociative analgesia was used by administering ketamine according to the doses adjusted by the anesthesia team. Fragmentation and localization of the stone was checked by using ultrasound and fluoroscopy during the procedure. The shock waves were initiated at level two to five for adult kidneys. After five hundred shock waves progressed the level was raised from level five to seven according to the fragmentation of stones observed on fluoroscopy and ultrasound. The rate of shock wave delivery is 90 shocks/minute.

The patients were reassessed after four weeks on ultrasound and X-ray KUB (kidney, ureter, and bladder) and if needed another ESWL session was arranged. The final appraisal of stone clearance was made at two to three months. If the fragments were not visualized on X-ray or ultrasound, the patients would have been declared completely as completely stone cleared. Nonobstructive or non-infected residual fragments of ≤4 mm were considered clinically inconsequential stone fragments and labeled as stone free in our study. Any patient who had undergone a major ancillary procedure after ESWL like percutaneous nephrolithotomy (PCNL) or ureterorenoscopy (URS) for stone clearance was labeled as a treatment collapse. We calculated the mean number of sessions, stone clearance rates, ancillary procedure rates, and complication rates. The data was obtained from the chart reviews of the patients. Statistical package for the social sciences (SPSS) version 16  (IBM Corporation, NY, USA) was used for data analysis. The mean ± standard deviation was calculated for quantitative variables like age of the patients, stone size, procedure time and hospital stay. Statistical analysis was performed using chi-square (*X^2^*) test for categorical variables like stone clearance rate. For continuous variables like some sessions of ESWL and number of shock waves, an independent sample t-test was used to compute P values.

## Results

The mean± standard deviation stone size was 1.28±61 cm in the adults and 1.08±0.59 cm in the children (Table 1). The post-ESWL stone clearance was 82/104 (79%) for the children and 204/300 (68%) for the adults (Table 2).

**Table 1:** Patient Demographics & Characteristics

Characteristics	Adult Group	Children Group
Number of patients	300	104
Males	214 (71.3%)	71 (68.24%)
Females	86 (28.7%)	33 (31.76%)
Age (Mean±SD)	40.22±1.57 years	7.84±4.22 years
Stone Size (Mean±SD)	1.28±61 cm	1.08±0.59cm

**Table 2:** Assessment of Outcomes *Chi-square (*X^2^) *test **Independent sample t-test

Outcomes Measured	Adult Group	Children Group	Significance
Stone free rate	204/300 (68%)	82/104 (79%)	P=0.036*
Number of shock waves (Mean±SD)	3525.33±491.83	2877.89±670.59	P=0.001**
Number of sessions (Mean±SD)	1.47±1.17	1.28±0.51	P=0.1097**

**Figure 2:**
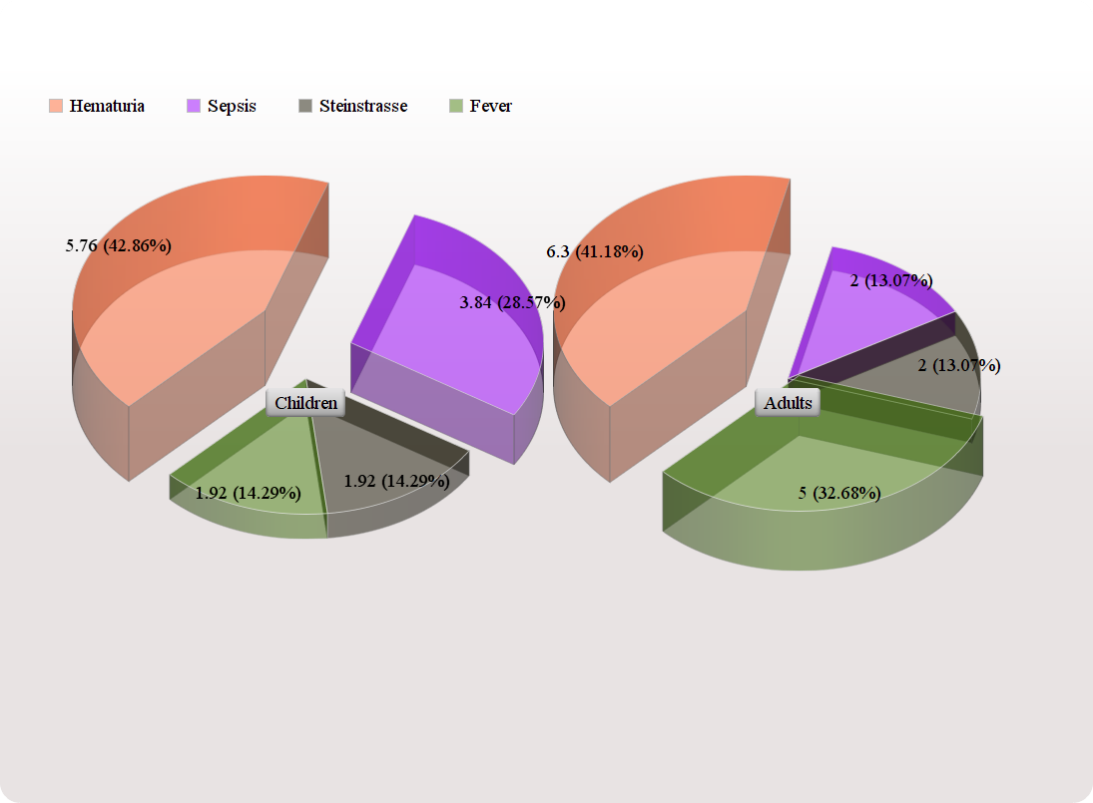
Post-ESWL Complications There is no statistical difference of complications between children and adults after extracorporeal shock wave lithotripsy treatment (*X^2^: *P>0.05)

In the children's group, a second session was required in 19/104 (18.2%) patients and the third session was required in 3/104 (2.8%) patients while in the adult group two sessions were required in 61 (20.1%) and three sessions were required in 24 (7.9%) patients. In the adults, the complications included steinstrasse in six patients (two percent), fever in 15 (five percent), hematuria in 19 (6.33%) and sepsis in six (two percent) patients. In the children, steinstrasse was found in two patients (1.92%), mild fever was diagnosed in two patients (1.92%), hematuria in six patients (5.76%) and sepsis was seen in four (3.84%) patients (Table 3) (Figure 2). The overall complication rate in the adults is 46/300 (15.33%) and in the children was 14/104 (13.46%). No statistical difference was found between post-ESWL complications between children and adults (P>0.05). Ancillary procedures including DJ stent were used in 13 patients (12.5%) in the children's group and in 78 patients (29%) in the adults group. A Chi-square (*X*^2^) test was applied to compare the stone clearance rate between the two groups and the P-value equals 0.036 which is statistically significant.

**Table 3:** Post-Extracoporeal Shock Wave Lithotripsy (ESWL) Complications

Complications	Adult Group	Children Group	P Value (*X^2^)*
Hematuria	19 (6.33%)	6 (5.76%)	1.0000
Sepsis	6 (2%)	4 (3.84%)	0.2887
Steinstrasse	6 (2%)	2 (1.92%)	1.000
Fever	15 (5%)	2 (1.92%)	0.2586

## Discussion

Renal and uretericstone fragmentation was done by extracorporeal shock wave lithotripsy in the children as well as in the adults. The factors affecting the stone-free rates are almost the same for both the pediatric and the adult population after extracorporeal shock wave lithotripsy treatment [12-13]. In our study, results that had no proof of residual fragments of size more than 4 mm on both ultrasound and X-ray were labeled as stone cleared. A result with the presence of any residual remains more than 4 mm in size was labeled as a treatment collapse. The patients with such stone fragments would form recurrent symptomatic stones that necessitate another ESWL treatment [14-15]. ESWL studies in the pediatric population have shown that 69% of patients would become symptomatic as residual fragments tend to mature [16]. A study by Tan, *et al*. reported only moderate efficacy of ESWL in the pediatric population [23]. He documented 100 patients with a mean stone size of 7.8 mm, where 60% of the patients were declared as stone cleared after the first extracorporeal shock wave lithotripsy session, and the stone-free rate amplified to 68% after a second session. In the present study, post-ESWL stone-free rate in the pediatric group is greater than that documented by Tan, et al., despite a larger mean stone size of 1.08 cm in the current study; this difference might be elaborated by the inclusion of multiple stones in the study of Tan, *et al.,* which has been excluded from our study [13]. 

In the current analysis, the stone clearance rate and re-ESWL session needed was comparable in children and adults. However, there was a unique variation between the numbers of shock waves required for stone clearance in the pediatric and adult groups in the current study. The mean stone size was 1.28±61 cm in the adults and 1.08 ± 0.59 cm in the children. The post-ESWL stone clearance was 82/104 (79%) in the children and 204/300 (68%) in the adults. In the children's group, a second session was required in 19/104 (18.2%) patients anda third session was required in 3/104 (2.8%) patients while in the adult group two sessions were required in 61 (20.1%) and three sessions in 24(7.9%) patients. Inthe adults, the complications included steinstrasse in six patients (1.9%), fever in 15 (4.9%), hematuria in 19 (6.2%) and sepsis in six (1.9%) patients, while in the children the complications were steinstrasse in two (1.9%) patients, mild fever in two (1.9%), hematuria in six (5.7%), and sepsis in four (3.8%) patients. The overall complication rate in the adultswas 46/300 (15%) and in the children was 14/104 (13%). Ancillary procedures including DJ stent were used in 13 patients (16%) in the children's group, while it was used in 29% of the adults. Extracorporeal shock wave therapy in the children was relatively complication-free in the present study, and similar results were depicted by Defoor W, et al. and Rhee K, et al. [17-18]. Steinstrasse was seen in six adults (1.9%) and two (1.9%) pediatric population undergoing ESWL in the present study. The complication rate regarding steinstrasse in the pediatric group in the present study is not seen in some studies that have documented the absence of steinstrasse after extracorporeal shock wave lithotripsy in children [18-19].However, in another study by Defoor W, et al., a similar rate of steinstrasse was seen in pediatric patients undergoing ESWL as was seen in our pediatric group [17]. 

An incidence of overall post-ESWL complications between pediatric and adult groups was not statistically significant in our study but, it has been marked that the children's group showed a clearance rate of 79% as compared to the adult group with a stone-free rate of 68% (P=0.036). However, there was no difference in some sessions for ESWL in the pediatric group as compared to adults (P=0.11). There has been a concern regarding the effects of shock waves delivered to immature kidneys in the pediatric group. It has been suggested that harmful effects of shock waves of ESWL can be avoided only by decreasing the number of shockwaves and reducing the energy in kilovolts [20]. Many studies have documented that there are no deleterious effects of ESWL on flourishing kidneys [21-23]. Considering the fact that we are still awaiting long-term data, it would seem prudent to shock the pediatric kidney as little as possible. But in the presence of ultrasonography and fluoroscopy stone-localization and stone-fragmentation monitoring can be done, that helps in decreasing the dose of harmful shock waves. 

### Limitations

It was a retrospective analysis based on a single-center experience. There is a limited external validity to this study, however the results of the study can not be ruled out. The results of this analysis cannot be generalized to other geographical regions of the world. The effect of difference in compositions of stones was not assessed between the two age groups which might have affected the outcome of ESWL.

## Conclusions

The results of ESWL for urolithiasis in adults remain inferior to that of children. Children can achieve high stone-free rates and have a lower need for repeat-ancillary procedures than adults. There is no difference in post-ESWL complications between children and adults.
